# Differences in resting state functional connectivity underlie visuomotor performance declines in older adults with a genetic risk (*APOE* ε4) for Alzheimer’s disease

**DOI:** 10.3389/fnagi.2022.1054523

**Published:** 2022-12-01

**Authors:** Alica Rogojin, Diana J. Gorbet, Kara M. Hawkins, Lauren E. Sergio

**Affiliations:** ^1^School of Kinesiology and Health Science, York University, Toronto, ON, Canada; ^2^Centre for Vision Research, York University, Toronto, ON, Canada; ^3^Vision Science to Applications (VISTA) Program, York University, Toronto, ON, Canada

**Keywords:** Alzheimer’s disease, apolipoprotein E4 (*APOE ε4*), dorsal attention network, default mode network, early detection, resting state functional connectivity, visuomotor integration

## Abstract

**Introduction:**

Non-standard visuomotor integration requires the interaction of large networks in the brain. Previous findings have shown that non-standard visuomotor performance is impaired in individuals with specific dementia risk factors (family history of dementia and presence of the *APOE* ε4 allele) in advance of any cognitive impairments. These findings suggest that visuomotor impairments are associated with early dementia-related brain changes. The current study examined the underlying resting state functional connectivity (RSFC) associated with impaired non-standard visuomotor performance, as well as the impacts of dementia family history, sex, and *APOE* status.

**Methods:**

Cognitively healthy older adults (*n* = 48) were tested on four visuomotor tasks where reach and gaze were increasingly spatially dissociated. Participants who had a family history of dementia or the *APOE* ε4 allele were considered to be at an increased risk for AD. To quantify RSFC within networks of interest, an EPI sequence sensitive to BOLD contrast was collected. The networks of interest were the default mode network (DMN), somatomotor network (SMN), dorsal attention network (DAN), ventral attention network (VAN), and frontoparietal control network (FPN).

**Results:**

Individuals with the ε4 allele showed abnormalities in RSFC between posterior DMN nodes that predicted poorer non-standard visuomotor performance. Specifically, multiple linear regression analyses revealed lower RSFC between the precuneus/posterior cingulate cortex and the left inferior parietal lobule as well as the left parahippocampal cortex. Presence of the *APOE* ε4 allele also modified the relationship between mean DAN RSFC and visuomotor control, where lower mean RSFC in the DAN predicted worse non-standard visuomotor performance only in *APOE* ε4 carriers. There were otherwise no effects of family history, *APOE* ε4 status, or sex on the relationship between RSFC and visuomotor performance for any of the other resting networks.

**Conclusion:**

The preliminary findings provide insight into the impact of *APOE* ε4-related genetic risk on neural networks underlying complex visuomotor transformations, and demonstrate that the non-standard visuomotor task paradigm discussed in this study may be used as a non-invasive, easily accessible assessment tool for dementia risk.

## Introduction

Alzheimer’s disease (AD) related brain changes are thought to begin several decades before the onset of clinical symptoms (Morris, 2005). Early identification of preclinical AD can allow for healthcare providers to make recommendations on lifestyle changes to mitigate modifiable risk factors such as diabetes, physical inactivity, and social isolation (Xu et al., 2015; Livingston et al., 2020; Litke et al., 2021) in order to delay or slow down disease progression. One of the important challenges facing AD clinical management today is the early detection or measure of small changes in behaviour or clinical signs in at-risk individuals that could accurately predict their likelihood of future disease-related impairment (Dubois et al., 2016). Current methods for detecting biomarkers of AD are expensive or invasive, such as abnormal amyloid, tau, or [18F]-fluorodeoxyglucose positron emission tomography (FDG-PET), atrophy in the brain using magnetic resonance imaging (MRI), and lumbar puncture for cerebrospinal fluid analysis (Rice and Bisdas, 2017; Jack et al., 2018; Shaw et al., 2018; Ou et al., 2019). Blood-based biomarkers are a more affordable option, however they are still invasive and require extensive laboratory resources (Nabers et al., 2018; Milà-Alomà et al., 2019; Otto et al., 2019). Behavioral measures that detect dysfunction of brain networks due to increased risk for AD may be a more affordable and easily accessible alternative to current diagnostic and risk assessment tools.

AD shows characteristic patterns of disruption to both structural (Filippi and Agosta, 2011; Gold et al., 2012; Shao et al., 2012; Peraza et al., 2019) and functional connectivity (Wang et al., 2007; Brier et al., 2012; Vemuri et al., 2012; Sheline and Raichle, 2013) between brain regions. In particular, the default mode network (DMN) has been a focus of AD biomarker research as regions of the default mode network largely overlap with regions showing earliest signs of AD pathology (Buckner et al., 2005; Palmqvist et al., 2017). Decreased functional connectivity between areas of the DMN has been demonstrated in cognitively healthy individuals with increased amyloid deposition (Hedden et al., 2009; Sheline et al., 2010), a family history of AD (Wang et al., 2012a), and presence of the *APOE* ε4 allele (Sheline et al., 2010; Wang et al., 2015; Liang et al., 2017). In addition to the DMN, other resting state functional networks have also been shown to have altered functional connectivity in preclinical AD. Cognitively normal older adults with amyloid burden have exhibited decreased functional connectivity in the frontoparietal control network (Elman et al., 2016; Buckley et al., 2017), dorsal attention network (Elman et al., 2016), and salience network (Buckley et al., 2017). Decreased functional connectivity was found in cognitively normal *APOE* ε4 carriers in the prefrontal cortex, cingulate cortex, and visual areas (McKenna et al., 2016), as well as in posterior regions of the DMN (Machulda et al., 2011). Other studies have reported increased functional connectivity within the salience network in older *APOE* ε4 carriers (Machulda et al., 2011; Liang et al., 2017). One study investigating resting functional connectivity with disease progression found initial hyperconnectivity in resting state networks in the very early stages of AD progression, followed by consistent reductions in functional connectivity in all networks with increasing AD severity (Brier et al., 2012).

One way to test the functional integrity of networks is to use a task that requires the integration of different domains (e.g., sensory, cognitive, and motor) and requires communication across multiple brain regions. Visuomotor tasks involving an element of dissociation or “decoupling” between the visual stimulus and target of the reach may present a novel behavioral target for dementia risk detection. Most everyday “standard” visually-guided arm movements involve a direct interaction with an object, such as picking up a mug of tea, and are automatic because the brain’s default visuomotor mapping is thought to have the gaze and hand spatially aligned (Gielen et al., 1984; Helsen et al., 1998). With the advent of tool use, humans can also perform decoupled or “non-standard” visually-guided arm movements that are more cognitively demanding as they must be learned or calibrated and involve the integration of some form of cognitive information into the movement (Wise et al., 1996). An example would be the increasingly common use of a rear-view camera (or the more traditional rear-view mirror) while backing up one’s car. To avoid an object, one needs to spin the wheel in a vertical plane leftward while viewing the object on the screen moving to the right; in this scenario the plane of limb motion is decoupled from guiding visual information, and there is a reversal of visual feedback. These more cognitively demanding non-standard tasks require sound connections between widespread brain regions within the frontoparietal network (Wise et al., 1997; Sabes, 2000; Filimon, 2010; Caminiti et al., 2017), and an inhibition of the default standard visuomotor control network (Gorbet et al., 2004). Two functional nodes of the DMN, the precuneus and inferior parietal lobule, are particularly important for discriminating between standard and non-standard task conditions (Gorbet and Sergio, 2016, 2018). Impairments in performance of increasingly dissociated visuomotor tasks have been reported in the early stages of AD-related dementia (Tippett et al., 2012; de Boer et al., 2014, 2015, 2016), in patients with mild cognitive impairment (Salek et al., 2011), and in cognitively healthy individuals at an increased risk for dementia (Hawkins and Sergio, 2014; Lu et al., 2021). Notably, these groups do not necessarily display large impairments in function when domains (sensory, cognitive, and motor) are tested individually.

Initial findings on the structural neural correlates underlying visuomotor impairments in *APOE* ε4 carriers without cognitive decline showed an association between reduced structural integrity in several major white matter tracts (Rogojin et al., in preparation). In the present study, we test the hypothesis that visuomotor impairments in cognitively healthy individuals with specific dementia risk factors (positive family history of dementia and presence of the *APOE* ε4 allele) may also be associated with reduced functional connectivity in the DMN, and altered functional connectivity in other resting state networks. These hypotheses were based on the (i) initial behavioral findings, (ii) known involvement of several DMN nodes in visuomotor integration, (iii) importance of the dorsal attention network, ventral attention network, frontoparietal network, and somatomotor network in aspects of visuomotor control (Corbetta et al., 2008; Wen et al., 2012; Tseng et al., 2013; Brovelli et al., 2015; Raichlen et al., 2016), and (iv) previously reported effects of APOE e4 on functional connectivity across multiple resting networks. As an exploratory component, we were also interested in exploring whether differences in functional connectivity of resting state networks may underlie previously reported sex-differences in visuomotor control (Rogojin et al., 2019) as sex-differences in resting brain connectivity have previously been demonstrated in the default mode, frontoparietal control, and sensorimotor networks (Zhang et al., 2018). Investigating the relationship between measures of network connectivity and visuomotor control in asymptomatic individuals with a genetic risk for AD will provide insight into the potential utility of a clinically accessible behavioral assessment of dementia risk.

## Materials and methods

### Participants

Forty-eight right-handed older adults with no cognitive impairments were included in the current study: 24 individuals with a positive family history of dementia (*n* = 11 females) and 24 individuals with no family history of dementia (*n* = 12 females; see Table 1 for demographic information). The current study used the same sample of participants as a previous behavioral study (Rogojin et al., 2019). All subjects scored at or above education-adjusted norms of 26 or higher on the Montreal Cognitive Assessment (MoCA), indicating no cognitive impairment. A positive family history of dementia was determined based on self-reported maternal or multiple family history (with at least one first-degree relative) of late-onset AD as a higher risk for AD is associated with a maternal or multiple, but not paternal, family history of dementia (Honea et al., 2010, 2011). Classification of no family history of dementia was based on having no family history of AD or any other type of dementia. Participants were excluded if their parents were deceased at a young age before dementia could be detected, or if they were estranged from either biological parent and did not know their medical history. Participants with a positive family history of dementia were age-balanced with those without a family history of dementia. The apolipoprotein E (*APOE*) ε4 allele is the only gene that has been consistently associated with late-onset AD, representing the best-established genetic risk factor for progression to clinical AD (Reitz and Mayeux, 2014; Dubois et al., 2016). Participants who were *APOE* ε4 carriers were considered to be at an increased risk for AD. The exclusion criteria included uncorrected visual impairments, upper-limb impairments or medical conditions that could hinder motor task performance (e.g., severe arthritis or dystonia), any neurological or psychiatric illnesses (e.g., Parkinson’s disease, depression, schizophrenia, alcoholism, and epilepsy), any history of head injury (e.g., mild and severe) or stroke, and medical diagnoses that might impact brain connectivity (i.e., hypertension or diabetes). The study protocol was approved by the Human Participants Review Sub-Committee of York University’s Ethics Review Board.

**Table 1:** Participant demographic features.

	*APOE* ε4 positive	*APOE* ε4 negative	FH+	FH−	Females	Males
Demographics						
n	15	31	24	24	23	25
Age (years)	59.8 ± 4.99	58.4 ± 5.99	58.8 ± 6.03	58.8 ± 5.29	58.7 ± 5.71	58.8 ± 5.63
Range	51–68	49–69	51–69	49–67	50–68	49–69
FH+	10 (67%)	14 (45%)			11 (48%)	13 (52%)
*APOE* ε4 positive			10 (67%)	5 (33%)	10 (43%)	5 (20%)
MoCA score	27.7 ± 1.44	28 ± 1.56	27.8 ± 1.48	28 ± 1.56	28.1 ± 1.51	27.6 ± 1.5
Range	26–30	26–30	26–30	26–30	26–30	26–30
Years of education	16.2 ± 3.78	17.7 ± 3.15	17.4 ± 3.52	16.8 ± 3.19	17.5 ± 3.06	16.7 ± 3.61
Range	11–23	12–24	11–23	12–24	12–24	11–22

FH+, positive family history of dementia; FH−, no family history of dementia; APOE, apolipoprotein E; MoCA, Montreal Cognitive Assessment.

### *APOE* genotyping

A total of 2 ml of saliva were collected from each subject in microtubes from Diamed Lab Supplies Inc. using collection aids from Cedarlane Labs. Saliva samples were sent to DNA Genotek Inc. (Ottawa, ON, Canada) for *APOE* genotyping. DNA was extracted from samples according to the manufacturer’s protocols. Genotyping for *APOE* involved single nucleotide polymorphism (SNP) genotyping, and samples were tested for SNPs rs429358 and rs7412. The proteins that are produced by the *APOE* gene are either E2, E3, or E4 combinations (for instance, E2/E3). For the current study, subjects were categorized as either having a presence (*APOE* ε4 positive) or absence (*APOE* ε4 negative) of the *APOE* ε4 allele. The breakdown of the *APOE* genotyping in female participants was as follows: ε3/ε3 (*n* = 12), ε3/ε4 (*n* = 11). The breakdown of the *APOE* genotyping in male participants was as follows: ε2/ε3 (*n* = 1), ε3/ε3 (*n* = 18), ε2/ε4 (*n* = 1), ε3/ε4 (*n* = 4). Due to inconclusive *APOE* genotyping results, one female and one male participant were excluded from statistical analyses looking at the *APOE* genotype.

### Behavioral data

#### Behavioral data acquisition

**Figure 1:**
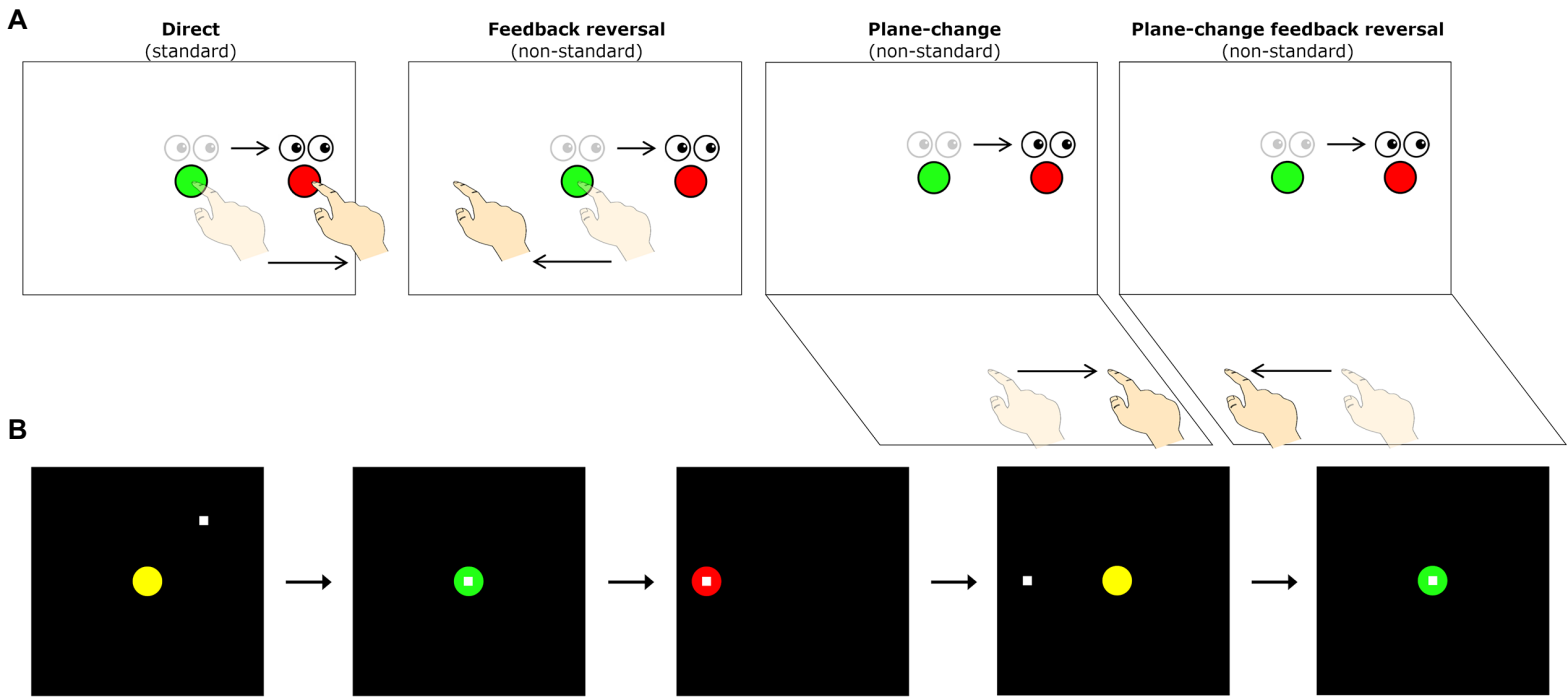
**(A)** Schematic drawing of the visuomotor task conditions. Lighter eye and hand symbols denote the starting position for each trial (green central target). Darker eye and hand symbols denote the instructed eye and hand movements for each task. Red circles denote the peripheral (reach) target, presented randomly in one of four locations (left, up, right, or down relative to the central target). The direct interaction task requires standard mapping, where participants slide their finger on a touch screen to move a cursor from a central target to one of four peripheral targets. The other three conditions involve non-standard visuomotor integration, where targets either have a 180° feedback reversal (feedback reversal), are spatially dissociated from the plane of hand motion (plane-change), or both (plane-change + feedback reversal). **(B)** Sequence of events during one trial of the visuomotor task. All trials begin in the central (home) target. Once the participant moves the cursor (denoted by the white square) into the central target, the target changes from yellow to green to signify a movement preparation period. After 4,000 ms, a red peripheral target appears in one of four directions (up, down, left, or right of the center) and serves as the ‘Go’ signal. Once the peripheral target is acquired and held for 500 ms it disappears, signaling the end of the trial. After an inter-trial interval of 2000 ms, the central yellow target reappears, and the participant moves back to the central target to initiate the next trial.

Our visuomotor assessment and behavioral data preprocessing have been described in detail in previous publications (Rogojin et al., 2019). Briefly, all subjects completed four visuomotor transformation tasks which involved making simple sliding finger movements between targets displayed on an Acer Iconia 6120 dual-touchscreen tablet. These tasks were divided into one standard condition (where gaze and movement were coupled) and three more cognitively demanding non-standard conditions (where gaze and movement were decoupled). Participants were instructed to slide the index finger of their right hand along the vertical or horizontal touchscreen (depending on the condition) in order to displace the cursor from a central target to one of four peripheral targets (to the top, bottom, left, or right of center) as quickly and as accurately as possible. In the standard mapping condition (S), the spatial location of the visual target and the required hand movement were the same. The non-standard mapping conditions required the finger movements to be made either on a different plane (plane-change, PC), in the opposite direction (feedback reversal, FR), or both (PC + FR), from the spatial peripheral target location (see Figure 1A for depictions of all four visuomotor transformation task conditions). Eye movements were the same across all conditions (i.e., always to the guiding visual peripheral target on the vertical screen). The four conditions were presented in randomized blocks, each consisting of five pseudo-randomly presented trials to each of the four peripheral targets for a total of 20 trials per condition, and thus 80 trials per participant across the four conditions. Each participant was also given two practice trials per peripheral target prior to each of the four conditions to ensure task comprehension. See Figure 1B for visual representations of a single trial completion, including trial timings.

#### Behavioral data preprocessing

Kinematic measures, including timing, finger position (x, y coordinates; 50 Hz sampling rate), and error data were recorded for each trial and converted into a MATLAB readable format using a custom written (C++) application. Custom analysis software (Matlab, Mathworks Inc.) was used to process individuals’ finger trajectories and generate movement profiles that were then verified by visual inspection, and manually corrected when necessary. All data were processed to compute timing, accuracy, and precision measures. These kinematic outcome measures were as follows: (1) Reaction time (RT), the time interval (in ms) between the central target disappearance and movement onset; (2) Full movement time (MTf), the time (in ms) between movement onset and offset; (3) Peak velocity (PV), the maximum velocity obtained during the ballistic movement, and used to calculate the 10% threshold used for determining movement onsets and offsets; (4) Path length (PL), the total distance (in mm, calculated from the x and y trajectories) travelled between movement onset and offset; (5) Absolute error (AE), a measure of end-point accuracy, and the average distance (in mm) from the individual ballistic movement endpoints (∑ x/n, ∑ y/n) to the actual target location; and (6) Variable error (VE), a measure of end-point precision, and the distance (in mm) between the individual ballistic movement endpoints (σ) from their mean movement. Corrective path length (CPL) represents corrective movements and was quantified by subtracting the PLb (initial movement offset) from the PLf (full movement offset). The procedure for combining some of the kinematic measures into composite scores to decrease the number of comparisons made in the data analysis was previously described (Rogojin et al., 2019). Briefly, all kinematic measures were standardized using z-scores and the composite scores were then created using simple averaging. A “timing score” was created as a composite score of RT, MTf, and PV, and an “endpoint error score” was a composite score of AE and VE. The timing and endpoint error scores had a high internal consistency, as indicated by Cronbach’s alpha values of 0.897 and 0.772, respectively. The corrective path lengths, timing scores, and endpoint error scores were then used for statistical analysis.

### Magnetic resonance imaging

#### Image acquisition

MRI data were acquired using a 3 Tesla (3 T) Siemens Trio scanner at York University. Participants received a T1-weighted anatomical scan using a sagittal volumetric magnetization-prepared rapid gradient echo (MP-RAGE) sequence. The MP-RAGE consisted of the following acquisition parameters: 192 sagittal slices (slice thickness of 1 mm, with no gap), field of view (FOV) of 256 × 256 mm, matrix size of 256 × 256 resulting in a voxel resolution of 1 × 1 × 1 mm, echo time (TE) = 2.96 ms, repetition time (TR) = 2,300 ms, flip angle = 9^o^. To quantify resting state functional connectivity (RSFC) within networks of interest, an echo planar imaging (EPI) sequence sensitive to blood oxygenation level dependent (BOLD) contrast was collected. Participants were asked to lie still with their eyes closed, letting their mind wander for 6 min in the scanner during the functional sequence with the following acquisition parameters: 35 axial slices (slice thickness of 4 mm, with no gap), field of view (FOV) of 210 × 210 mm, matrix size of 70 × 70 resulting in a voxel resolution of 3 × 3 × 4 mm, echo time (TE) = 30 ms, repetition time (TR) = 2000 ms, flip angle = 90^o^.

#### MR image preprocessing

Preprocessing of the neuroimaging data was done using FreeSurfer 7.1 (Harvard Medical School, Boston, United States; http://www.surfer.nmr.mgh.harvard.edu) and Analysis of Functional NeuroImages (AFNI) software (version 21.3.07 “Trajan”; Cox, 1996). The T1-weighted MR images were used as input to the main Freesurfer reconstruction pipeline (“*recon-all*”) to parcellate and segment the brain into anatomically distinct regions of the cortex and subcortical nuclei, respectively. Each individual’s parcellation and segmentation was visually inspected to ensure that there were no obvious errors. AFNI was used for preprocessing of the resting state data. For every participant, the first 2 image-volumes were removed. Functional preprocessing steps included despiking (3dDespike), slice timing correction (3dTshift), coregistration of the rest run with the subject’s T1-weighted anatomical image (3dAllineate), and motion correction (3dVolreg). ANATICOR (Jo et al., 2010) was used for nuisance variable regression (removal of ventricle/white matter tissue-based signals). The ventricle and white matter masks used for nuisance regression by ANATICOR were created from Freesurfer’s segmentation of the T1-weighted data and were visually-inspected for quality control. Within AFNI, these masks were eroded to prevent inclusion of voxels containing gray matter. Motion censoring was applied such that successive functional volumes containing >0.25 mm of head motion, as well as volumes containing >5% of voxels as outliers, were removed. At each voxel, the data was detrended using the default polynomial degree based on the duration of the resting state run. A voxel was labeled an outlier if the signal was outside of a defined number of mean absolute deviations (MAD) from the trend, calculated based on the number of TRs in the dataset. To account for older adults’ tendency to move more in the scanner (Pardoe et al., 2016; Savalia et al., 2017; Madan, 2018), the motion censor threshold used in the current study was less conservative than that typically used in resting state fMRI preprocessing pipelines for younger participants (>0.2 mm; Power et al., 2014). Average motion after censoring was <0.15 mm for all participants. The percentage of censored volumes for each group was as follows: family history positive (6.7%) vs. no family history (6.1%), males (7.6%) vs. females (5.1%), *APOE* ε4 positive (12.5%) vs. *APOE* ε4 negative (3.2%). The percentage of censored volumes and average censored motion did not differ significantly (*p* > 0.05) between groups (family history positive vs. no family history, males vs. females, *APOE* ε4 positive vs. *APOE* ε4 negative). In addition to nuisance variables created *via* ANATICOR, nuisance variables for the time-series of each voxel also included estimates of head motion in six directions and the temporal derivative of each of these six head motion estimates. Least-squares model fitted time-series of all nuisance variables were subtracted from the voxel time-series resulting in a residual time-series that was used in subsequent analyses. As a data quality assessment procedure, the precuneus was used as a seed region in the preprocessed functional data (i.e., the residual time-series) in order to verify that the DMN looked reasonable in every subject. The seed region was selected within the left hemisphere precuneus, 2–3 slices away from the mid-sagittal plane, central to the region bordered by the pars marginalis of the cingulate sulcus, the subparietal sulcus, and the parieto-occipital fissure. Following preprocessing, one participant was excluded due to missing resting state data, and 48 participants were used in the final analysis.

#### Functional connectivity parcellation

An individualized functional parcellation approach was used to identify subject-specific functional resting state network nodes in the preprocessed data (Chong et al., 2017). Functional parcellation of resting state networks refers to the identification of cortical areas, or ‘parcels’ that exhibit functionally similar properties (Raichle et al., 2001; Sporns et al., 2005; Kim et al., 2010; Eickhoff et al., 2011, 2015; Smith et al., 2013). The most common approach to parcellation relies on a mean functional resting state network parcellation common to a group of subjects (i.e., the group average network), which is then projected back onto individual subject data (Thomas Yeo et al., 2011; Wig et al., 2014). These population-average networks have provided important information on the large-scale functional organization of the brain (Buckner et al., 2013; Wig, 2017). However, population-average networks may obscure individual-specific network organization and thus lead to inaccuracies at the level of individual subjects (Chong et al., 2017). Thus, there is a growing focus on person-specific parcellation to define functional parcels independently for each participant. Group prior individual parcellation (GPIP) was implemented to automatically perform parcellation of resting functional data into functional networks at the level of individual subjects (Chong et al., 2017). GPIP is a novel cortical parcellation method that initializes parcellation using an atlas template. This initial parcellation is then refined for each subject using the subject’s functional data to allow individual variability across subjects in the boundaries of these parcels (Chong et al., 2017). The use of a template atlas for parcellation initialization results in all subjects having corresponding functional regions (aiding group analysis), while functional parcel boundaries can vary from subject to subject. GPIP iterates between two steps to continuously update parcel labels until convergence: (1) each participant’s parcel boundaries (first obtained from the initialization to the Schaefer atlas) are refined relative to their resting functional data, and (2) the concentration (inverse covariance/partial correlation) matrices from all individuals are then jointly estimated using a group sparsity constraint (Chong et al., 2017). Specifically, for the results presented in the current study, the preprocessed and denoised resting state functional data were first initialized with the 200-parcel Schaefer atlas (Schaefer et al., 2018), corresponding to the 7 functional networks atlas (Thomas Yeo et al., 2011).

**Figure 2:**
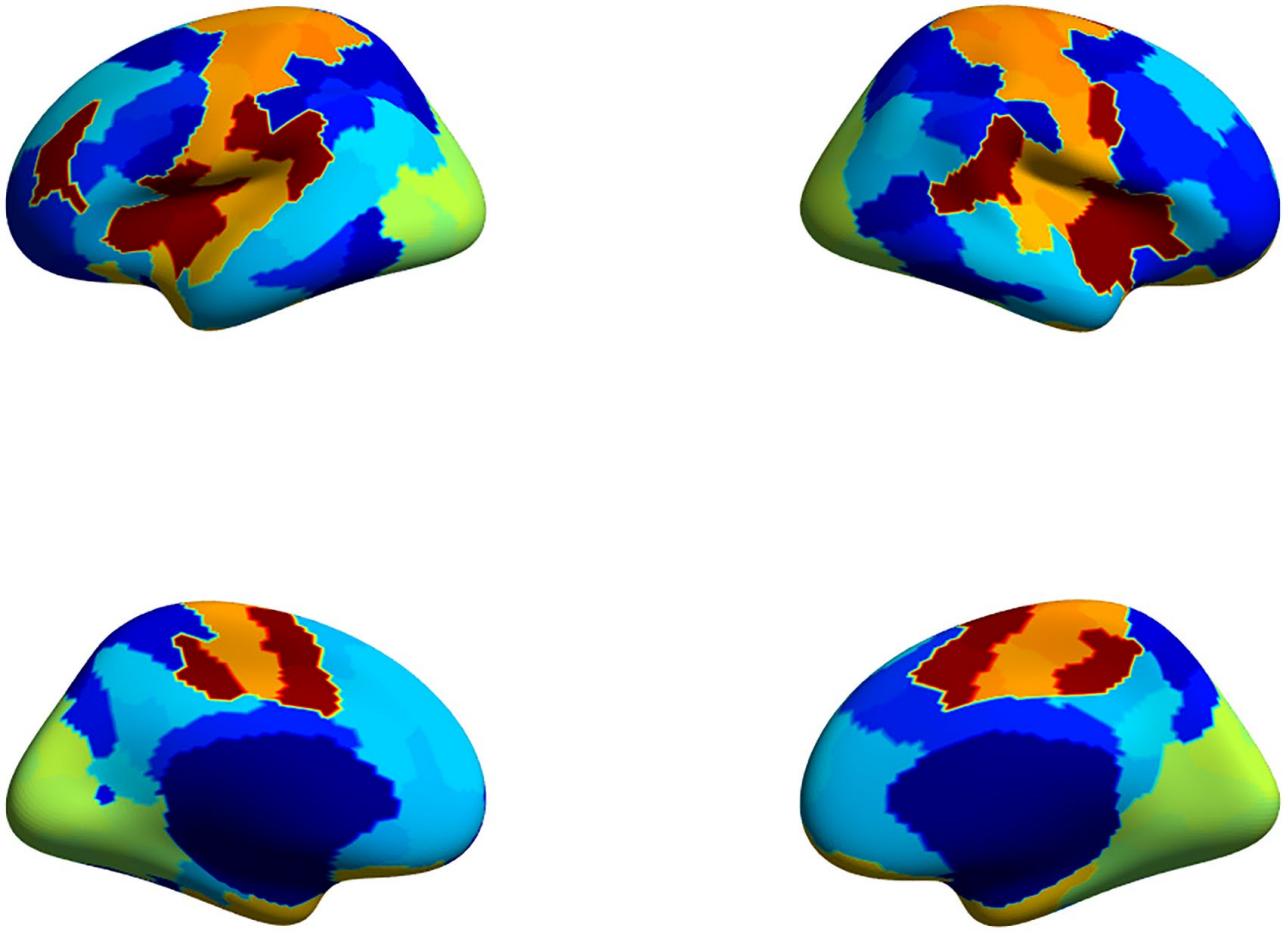
Example parcellation from a single subject initialized with the 200-parcel 7-network Schaefer atlas (Schaefer et al., 2018) with refined parcel borders that functionally correspond to the subject’s resting state data as a result of the GPIP process.

Prior to GPIP initialization, the functional data were registered to the corresponding FreeSurfer anatomical images for each subject, converted from volumetric to surface space, and resampled to the FreeSurfer cortical surface template (fsaverage5). Spatial smoothing of 6 mm was applied to the anatomically-aligned data in surface space. Visual inspection was used to verify proper coregistration of functional data with the T1-weighted anatomical images. Values from vertices located in the medial wall were resampled into the surface data as they are removed by FreeSurfer but are needed for running GPIP. The functional time-series data were normalized by scaling to a mean value of 0 and a standard deviation of 1. The normalized functional time-series data were then used in subsequent steps for GPIP analysis. GPIP performed its two-step iterative process 20 times for each subject resulting in increasingly refined functional network parcellations with optimal segmentation with respect to the cortical surface. Each participant’s final parcellation was plotted and inspected to verify the quality of the parcellation (an example of a single subject’s final parcellation is shown in Figure 2). To further assess the quality of the parcellations, homogeneity was calculated as the mean temporal correlation coefficient between all pairs of vertices within each GPIP parcel, where a large value suggests that the vertices included in a particular parcel have similar time-series (i.e., are homogeneous) and therefore correctly assigned to that parcel. The homogeneity value was calculated for the whole brain as a mean value across all parcels for each GPIP iteration to verify that these values increased with each iteration before plateauing prior to the final iterations, suggesting stable and accurate parcellations. Further, cross-correlation matrices including all GPIP parcels were plotted and visually inspected to verify reasonable patterns of whole-brain connectivity in each participant (see section “Resting state functional connectivity matrix” below for specific details on matrix construction).

#### Resting state functional connectivity matrix

**Figure 3:**
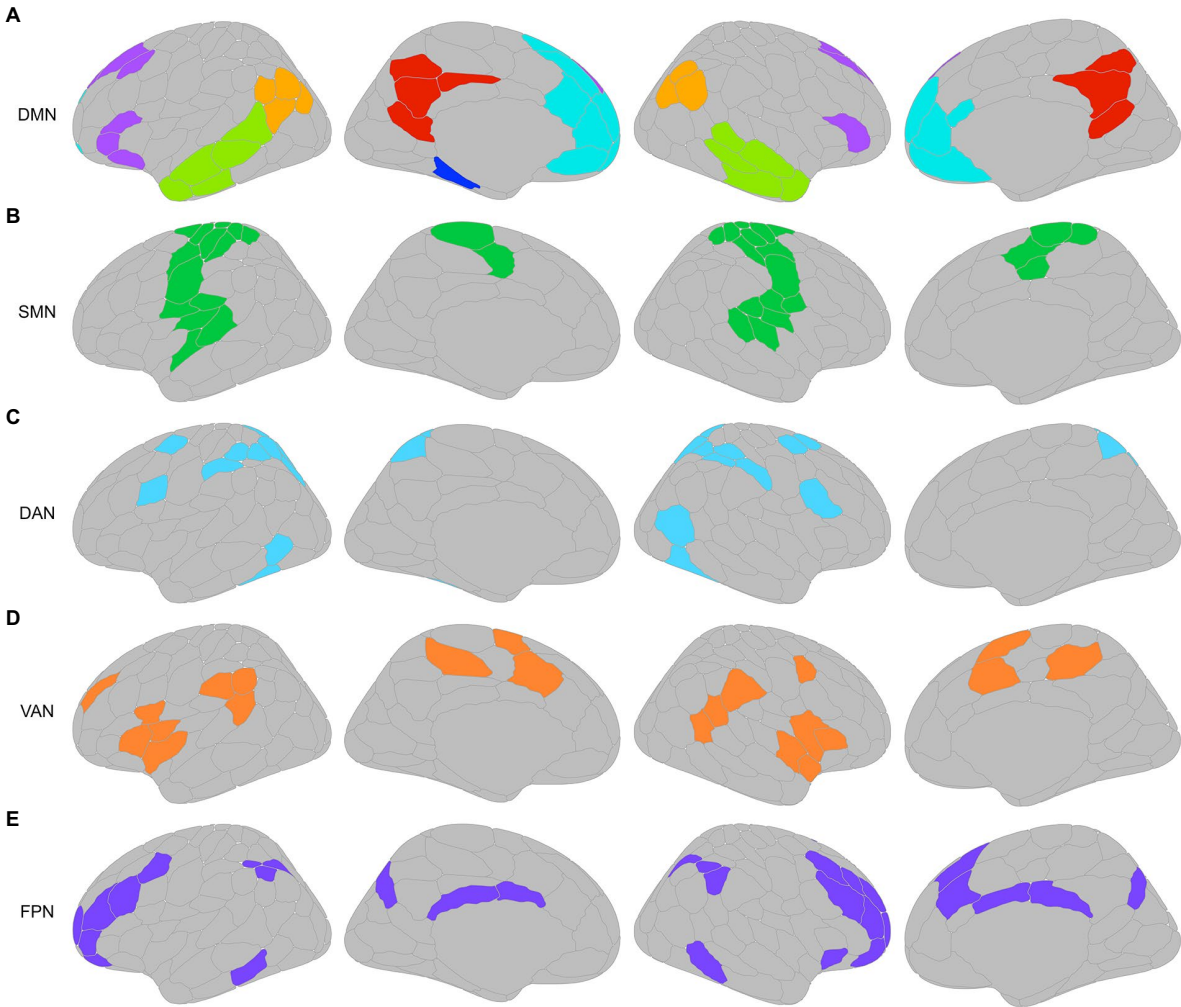
**(A)** Parcels from GPIP parcellation of the default mode network (DMN) were visually inspected and assigned to the following DMN nodes: precuneus/posterior cingulate cortex (pCun/PCC; red), medial prefrontal cortex (mPFC; cyan), left and right lateral prefrontal cortex (llPFC, rlPFC; purple), left and right inferior parietal lobe (lIPL, rIPL; orange), left and right lateral temporal cortex (lLTC, rLTC; lime green), and left parahippocampal cortex (lPHC; dark blue). Resting state networks used for mean resting state functional connectivity (RSFC) analysis, with corresponding nodes assigned by GPIP to the **(B)** somatomotor network (SMN; green), **(C)** dorsal attention network (DAN; blue), **(D)** ventral attention network (VAN; orange), and **(E)** frontoparietal control network (FPN; purple). Brain figures made with “ggseg” R package (Mowinckel and Vidal-Piñeiro, 2020).

A functional connectivity matrix was created for each participant based on their individualized parcellation. The mean BOLD signal time-series data was extracted from each parcel and pairwise correlations were computed between each parcel pair. The correlation coefficients were then converted to z-scores using Fisher’s r-to-z transformation to normalize the distribution of correlation values, resulting in a 200 × 200 functional connectivity matrix for each subject. A growing body of research has implicated the DMN as being particularly vulnerable to AD pathology beginning in the preclinical stage (Hedden et al., 2009; Sheline et al., 2010; Sheline and Raichle, 2013; Riedel et al., 2016; Hampton et al., 2020). Functional nodes of the DMN include the posterior cingulate cortex, retrosplenial cortex, precuneus, medial prefrontal cortex, inferior parietal lobes, temporal pole extending into the lateral temporal cortex, and regions of the medial temporal lobe (including the hippocampal and parahippocampal cortices; Parvizi et al., 2006; Uddin et al., 2009; Andrews-Hanna et al., 2010; Sperling et al., 2011; Márquez and Yassa, 2019). Previous studies have further identified two functional cores of the DMN: the medial prefrontal cortex within the anterior medial portion of the DMN, and the precuneus and posterior cingulate cortex in the posterior medial portion of the DMN (Buckner et al., 2008; Fransson and Marrelec, 2008; Andrews-Hanna et al., 2010; Utevsky et al., 2014). These regions represent core hubs to which all other regions of the DMN are correlated (Buckner et al., 2008). As previously mentioned, functional connectivity in the DMN is disrupted in preclinical AD including prominently in the posterior medial regions (precuneus and posterior cingulate cortex) and the medial prefrontal cortex (Hedden et al., 2009; Damoiseaux et al., 2012; Wang et al., 2012b; Riedel et al., 2016), as well as between posterior medial regions and other posterior areas of the DMN, namely the medial temporal lobe and inferior parietal lobule (Sheline et al., 2010; López-Sanz et al., 2017). Therefore, instead of examining the DMN as a whole, homogeneous network, it was assessed based on the functional connectivity between and within the anterior and posterior core hubs, as well as the connectivity of these regions to other functional nodes within the DMN. The Schaefer parcellation was visually inspected to assign GPIP parcels of the DMN to the following network nodes: precuneus/posterior cingulate cortex (pCun/PCC), medial prefrontal cortex (mPFC), left and right lateral prefrontal cortex (llPFC and rlPFC), left and right inferior parietal lobe (lIPL, rIPL), left and right lateral temporal cortex (lLTC and rLTC), and left parahippocampal cortex (lPHC; Figure 3A). The IPL consisted largely of the angular and supramarginal gyri, and the LTC consisted of the temporal pole extending into the inferior, middle, and superior temporal gyri. Each participant’s mean Fisher z-transformed RSFC values were also extracted for several other resting state networks of interest as a measure of overall within-network functional connectivity. The networks of interest were the somatomotor network (SMN), dorsal attention network (DAN), ventral attention network (VAN), and frontoparietal control network (FPN; Figures 3B–E).

### Statistical analysis

All statistical analyses were carried out using open-source R software v4.1.0 (R Core Team, 2021). All participant groups were age-balanced, with no statistically significant differences in age observed between individuals with vs. without a family history of dementia (*p* = 0.87), *APOE* ε4 positive vs. ε4 negative participants (*p* = 0.64), and female vs. male participants (*p* = 0.82). There were also no statistically significant differences observed between groups on MoCA scores (FH+ vs. FH− *p* = 0.58; ε4+ vs. ε4− *p* = 0.74; female vs. male *p* = 0.20), years of education (FH+ vs. FH− *p* = 0.57; ε4+ vs. ε4− *p* = 0.15; female vs. male *p* = 0.54), computer experience (FH+ vs. FH− *p* = 0.36; ε4+ vs. ε4− *p* = 0.51; female vs. male *p* = 0.46), and touchscreen experience (FH+ vs. FH− *p* = 0.92; ε4+ vs. ε4− *p* = 0.62; female vs. male *p* = 0.55).

#### Family history of dementia, *APOE* status, and sex effects on functional connectivity

Three-way ANOVAs were used to compare differences in RSFC between: (1) individuals with a family history of dementia and individuals without a family history of dementia, (2) *APOE* ε4 carriers and non-carriers, and (3) females and males. The RSFC measures being compared were pairwise parcel FC values for select DMN nodes, as well as mean within-network RSFC in the SMN, DAN, VAN, and FPN. The pairwise comparisons for the DMN were performed between the following nodes: pCun/PCC and mPFC; pCun/PCC and bilateral lPFC, bilateral IPL, bilateral LTC, and left PHC; mPFC and bilateral lPFC, bilateral IPL, bilateral LTC, and left PHC. Post-hoc analyses were adjusted for multiple comparisons using the Holm correction method (Holm, 1979) across all models, as well as at the level of each regression analysis, and were considered statistically significant at an alpha of *p* < 0.05.

#### Family history of dementia, *APOE* status, sex, and functional connectivity effects on visuomotor integration performance

Previous behavioral findings revealed that significant predictors of worse visuomotor performance were (1) a family history of dementia (greater endpoint error scores in the visual feedback reversal condition), (2) having the *APOE* ε4 allele (greater endpoint error scores in the plane-change feedback reversal condition, and greater corrective path lengths in both of the plane-change conditions), and (3) sex (greater endpoint error scores in the visual feedback reversal condition; Rogojin et al., 2019). The current study used multiple linear regression analysis to assess whether these performance declines are associated with RSFC. The RSFC measures being compared were pairwise parcel FC values for select DMN nodes, as well as for mean within-network FC for the SMN, DAN, VAN, and FPN. The pairwise comparisons for the DMN were performed between the following nodes: pCun/PCC and mPFC; pCun/PCC and bilateral lPFC, bilateral IPL, bilateral LTC, and left PHC; mPFC and bilateral lPFC, bilateral IPL, bilateral LTC, and left PHC. Regression models were set up with RSFC as the predictor variable, one of the visuomotor performance measures as the dependent variable, and based on the previous findings they were controlled for (1) sex and family history in the feedback reversal condition, and (2) *APOE* ε4 status in the two plane-change conditions. Specifically, the visuomotor performance measures used in this study were endpoint error scores in the feedback reversal condition (controlled for sex and family history), endpoint error scores in the plane-change feedback reversal condition (controlled for *APOE* status), anßd corrective path lengths in both of the plane-change conditions (controlled for *APOE* status). The *p*-values were adjusted for multiple comparisons using the Holm correction method (Holm, 1979) applied across all models, as well as at the level of each regression analysis. Corrections were considered statistically significant at *p* < 0.05. All significant interaction findings reported below were replicated using robust linear regression models to ensure that results were not driven by outliers.

## Results

### No general effects of family history of dementia, *APOE* status, and sex on functional connectivity

There were no statistically significant differences between (1) positive and negative dementia family history, (2) *APOE* ε4 carriers and non-carriers, and (3) females and males found between pairwise parcel FC values for the DMN nodes of interest, or for mean within-network FC in the SMN, DAN, VAN, and FPN.

### Modifying effects of the *APOE* ε4 allele on the relationship between visuomotor performance and connectivity between the posterior medial DMN with other DMN nodes

**Figure 4:**
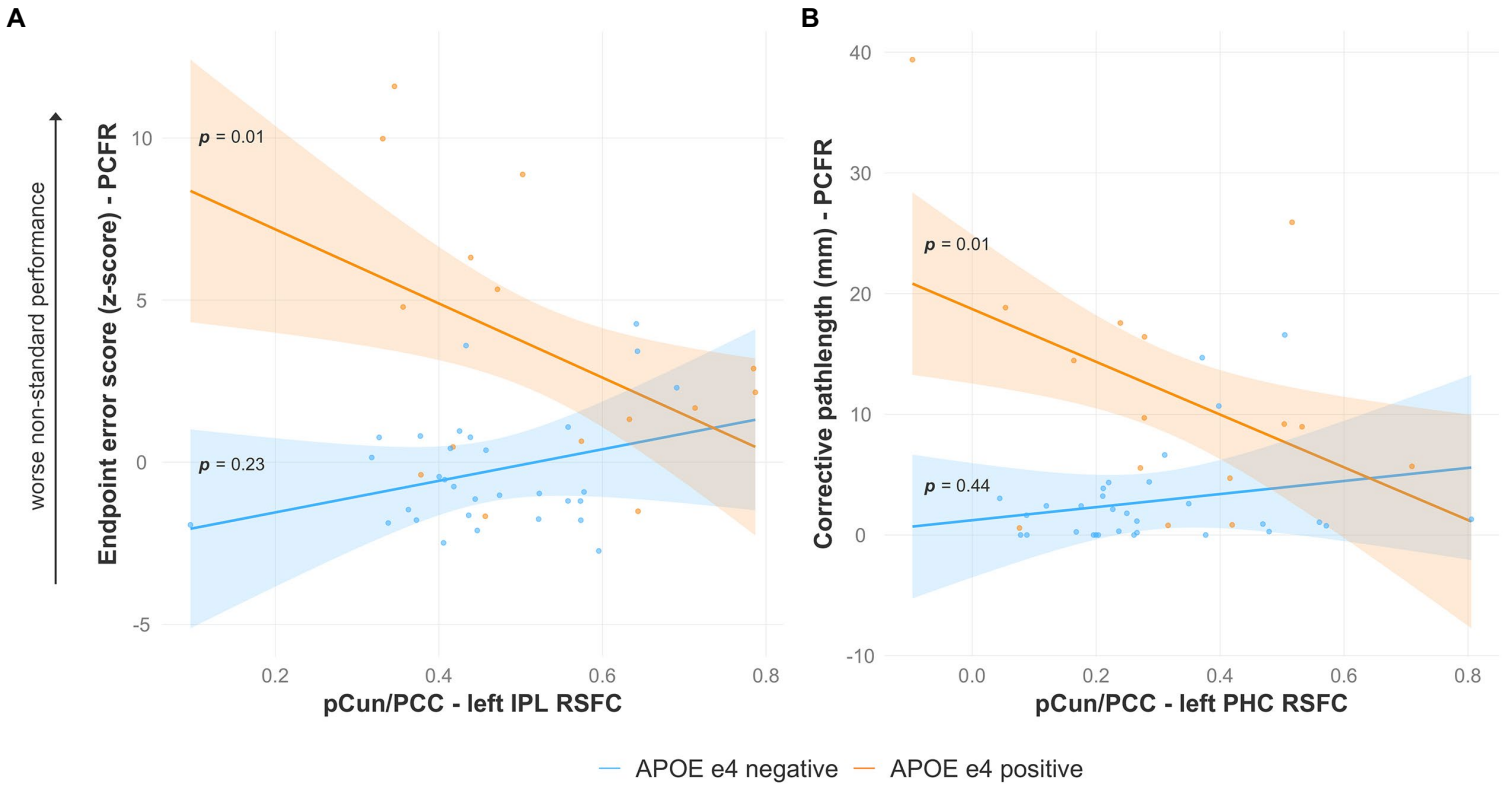
Lower RSFC between several DMN nodes predicts worse visuomotor performance (reflected by higher endpoint error scores or corrective path lengths) in the plane-change feedback reversal condition only in *APOE* ε4 carriers. Specifically, lower RSFC between the **(A)** pCun/PCC and left IPL, and **(B)** pCun/PCC and left PHC predicts worse non-standard visuomotor performance. Significant results shown from multiple regression and simple slopes analyses. RSFC, resting state functional connectivity; *APOE*, apolipoprotein E; pCun/PCC, precuneus/posterior cingulate cortex; IPL, inferior parietal lobule; PHC, parahippocampal cortex; PC + FR, plane change feedback reversal condition.

Presence of the *APOE* ε4 allele modified the relationship between visuomotor performance in the plane-change feedback reversal condition and RSFC between the pCun/PCC and two other nodes in the left hemisphere (Table 2). There was a significant interaction effect of pCun/PCC to left IPL RSFC and *APOE* status (β = −16.303, *p* < 0.05) on endpoint error scores (Figure 4A). The results of the regression analysis indicated that the predictors explained 37% of variance (R^2^_adj_ = 0.3696, F_3,42_ = 9.796, *p* < 0.01). Simple slopes analysis revealed that a lower RSFC between the pCun/PCC and left IPL (Figure 3A, red/orange) was a significant predictor of greater endpoint error scores (indicative of worse visuomotor performance) only in *APOE* ε4 carriers (simple slope = −11.43, S.E. = 4.43, *p* = 0.01). Similarly, there was a significant interaction effect of pCun/PCC to left PHC RSFC and *APOE* status (β = −27.278, *p* < 0.05) on corrective path length (Figure 4B). The results of the regression analysis indicated that the predictors explained 35.3% of variance (R^2^_adj_ = 0.3532, F_3,42_ = 9.192, *p* < 0.01). Simple slopes analysis revealed that a lower RSFC between the pCun/PCC and left PHC (Figure 3A, red/dark blue) was a significant predictor of greater corrective path length (indicative of worse visuomotor performance) only in *APOE* ε4 carriers (simple slope = −21.86, S.E. = 8.20, *p* = 0.01).

**Table 2:** Multiple linear regression models used to assess whether non-standard visuomotor performance declines are associated with resting state functional connectivity (RSFC).

Outcome	Network	Predictor	Estimate	S.E.	*t*	Unadjusted *p*	Adjusted *p*
EE Score	pCun/PCC –	Intercept	3.474	1.531	2.268	0.028508	
	left IPL						
		RSFC	−3.283	2.979	−1.102	0.276775	0.276775
		*APOE*	11.991	3.063	3.915	0.000326	0.000978
		RSFC*APOE	−16.303	5.958	−2.736	0.009068	0.018136
R^2^_adj_ = 0.3696, F_3,42_ = 9.796, adjusted *p* = 0.00470673 (unadjusted *p* = 0.00005061)							
*APOE* ε4 simple slope = −11.43, S.E. = 4.43, *p* < 0.01							
CPL	pCun/PCC –	Intercept	9.972	1.927	5.174	6.03E-06	
	left PHC						
		RSFC	−8.219	5.397	−1.523	0.1353	0.1353
		*APOE*	17.498	3.854	4.54	4.67E-05	0.0001401
		RSFC*APOE	−27.278	10.795	−2.527	0.0154	0.0308
R^2^_adj_ = 0.3532, F_3,42_ = 9.192, adjusted *p* = 0.00784852 (unadjusted *p* = 0.000008531)							
*APOE* ε4 simple slope = −21.86, S.E. = 8.20, *p* < 0.01							
EE Score	Mean DAN	Intercept	6.41	1.934	3.314	0.001898	
		RSFC	−10.638	4.168	−2.552	0.014422	0.014422
		*APOE*	15.345	3.868	3.967	0.000278	0.000834
		RSFC*APOE	−25.543	8.336	−3.064	0.003802	0.007604
R^2^_adj_ = 0.4148, F_3,42_ = 11.63, adjusted *p* = 0.00105545 (unadjusted *p* = 0.0000111)							
*APOE* ε4 simple slope = −23.41, S.E. = 6.81, *p* < 0.01							
CPL	Mean DAN	Intercept	18.08	4.895	3.694	0.000632	
		RSFC	−23.771	10.547	−2.254	0.02949	0.02949
		*APOE*	35.948	9.789	3.672	0.000674	0.002022
		RSFC*APOE	−59.268	21.094	−2.81	0.007498	0.014996
R^2^_adj_ = 0.3799, F_3,42_ = 10.19, adjusted *p* = 0.00340562 (unadjusted *p* = 0.00003623)							
*APOE* ε4 simple slope = −53.40, S.E. = 17.23, p < 0.01							

Regression models were set up with RSFC as the predictor variable, *APOE* ε4 status as the moderator variable, and visuomotor performance measures (EE score and CPL) in the plane-change feedback reversal condition as the outcome variable. Models with significant interactions are shown. Unadjusted and adjusted p-values reported. Values were adjusted for multiple comparisons using the Holm correction method and were considered statistically significant at *p* < 0.05. S.E., standard error; EE score, endpoint error score; CPL, corrective path length; RSFC, resting state functional connectivity; APOE, apolipoprotein E; DAN, dorsal attention network; pCun/PCC, precuneus/posterior cingulate cortex; IPL, inferior parietal lobule; PHC, parahippocampal cortex.

### Modifying effects of the *APOE* ε4 allele on the relationship between mean intra-DAN functional connectivity and visuomotor performance

**Figure 5:**
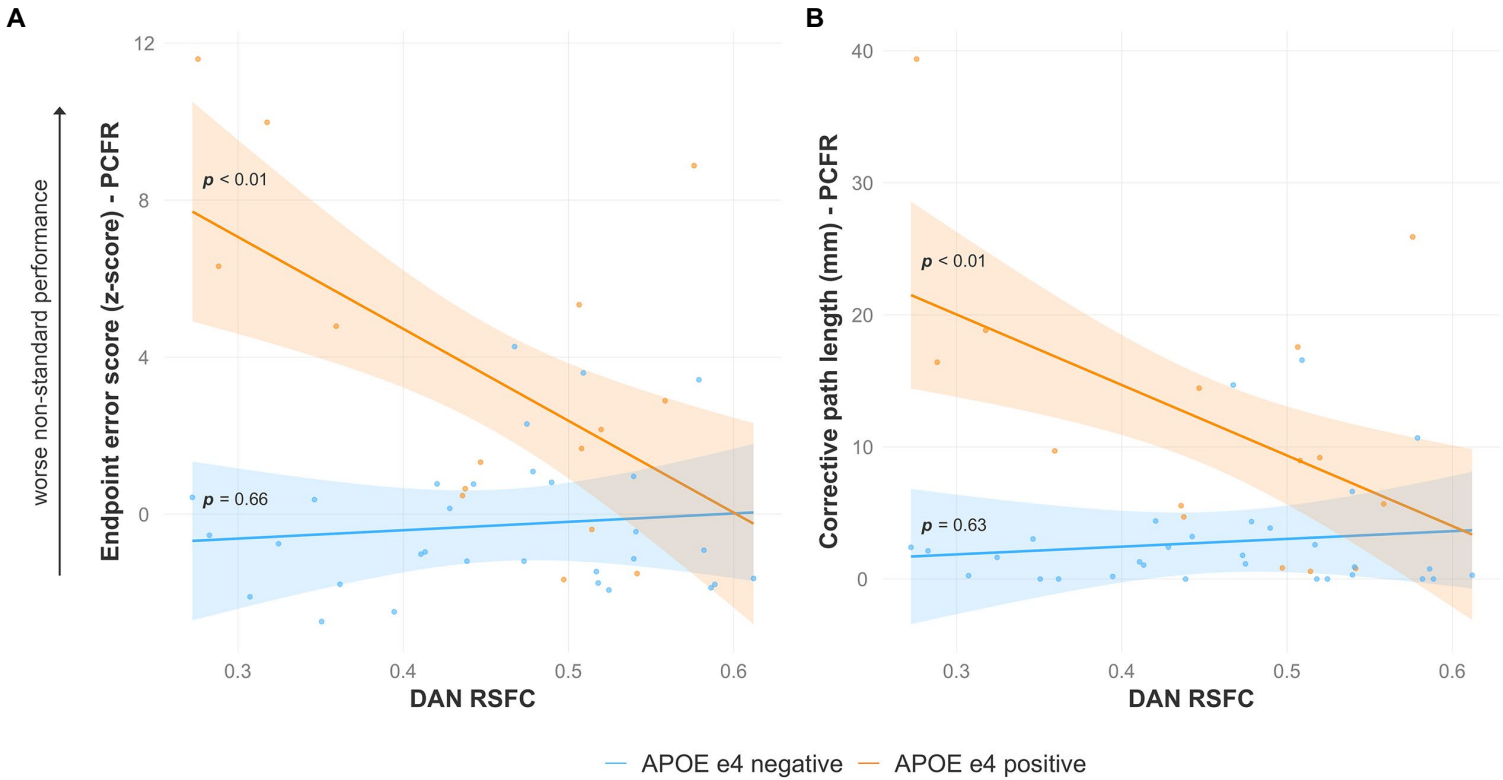
Lower intra-DAN mean RSFC predicts worse visuomotor performance in the plane-change feedback reversal condition only in *APOE* ε4 carriers. Worse non-standard visuomotor performance is reflected by **(A)** higher endpoint error scores, and **(B)** greater corrective path lengths. Significant results shown from multiple regression and simple slopes analyses. RSFC, resting state functional connectivity; *APOE*, apolipoprotein E; DAN, dorsal attention network; PC + FR, plane change feedback reversal condition.

Presence of the *APOE* ε4 allele modified the relationship between mean intra-DAN RSFC and visuomotor performance for two of the behavioral measures in the plane-change feedback reversal condition (Figure 5). There was a significant interaction effect of mean RSFC in the DAN and *APOE* status (β = −25.543, *p* < 0.01) on endpoint error scores. The results of the regression analysis indicated that the predictors explained 41.5% of variance (R^2^_adj_ = 0.4148, F_3,42_ = 11.63, *p* < 0.01). Simple slopes analysis revealed that a lower mean RSFC in the DAN was a significant predictor of greater endpoint error scores (indicative of worse visuomotor performance) only in *APOE* ε4 carriers (simple slope = −23.41, S.E. = 6.81, *p* < 0.01). Similarly, there was also a significant interaction effect of mean RSFC in the DAN and *APOE* status (β = −59.268, *p* < 0.05) on corrective path length. The results of the regression analysis indicated that the predictors explained 38% of variance (R^2^_adj_ = 0.3799, F_3,42_ = 5.915, *p* < 0.01). Simple slopes analysis revealed that a lower mean RSFC in the DAN was a significant predictor of greater corrective path length (indicative of worse visuomotor performance) only in *APOE* ε4 carriers (simple slope = −53.40, S.E. = 17.23, *p* < 0.01). The statistics for these multiple linear regressions are listed in Table 2. There were otherwise no effects of family history, *APOE* ε4 status, or sex on the relationship between mean within-network functional connectivity and visuomotor performance for any of the other resting networks.

## Discussion

The main goal of this study was to assess whether disrupted RSFC may underlie impaired visuomotor abilities in individuals with specific dementia risk factors (positive family history of dementia, or presence of the *APOE* ε4 allele). A secondary goal was to investigate whether differences in RSFC may also explain previously demonstrated sex-differences in non-standard visuomotor performance. We previously found that ε4 carriers performed significantly worse on visuomotor tasks under increasing cognitively-demanding conditions (Rogojin et al., 2019), and that this impaired performance was associated with lower white matter integrity in the brain (Rogojin et al., in preparation). Our key novel findings in the current study demonstrate that the ε4 allele also modified the relationship between RSFC and visuomotor performance, where lower RSFC in the DMN and the DAN predicted poorer non-standard performance. Notably, these visuomotor deficits in *APOE* ε4 carriers were detectable using our non-standard visuomotor tasks *in advance of any measurable cognitive issues*. There were no significant relationships between RSFC and family history of dementia or sex to explain previously reported differences in visuomotor task performance. There were also no main effects of family history, sex, or *APOE* ε4 carrier status on RSFC on any of the networks of interest.

### Reduced resting state functional connectivity in the posterior DMN and the DAN predict visuomotor deficits in *APOE* ε4 carriers

Individuals with the ε4 allele showed abnormalities in RSFC between posterior DMN nodes that predicted poorer non-standard visuomotor performance. Specifically, there was lower RSFC between the precuneus and posterior cingulate cortex, the posterior functional core of the DMN (Buckner et al., 2008; Fransson and Marrelec, 2008; Andrews-Hanna et al., 2010; Utevsky et al., 2014), and the left IPL and left parahippocampal cortex. Our findings are consistent with the literature, where reduced functional connectivity was demonstrated in the DMN between the precuneus and left parahippocampal cortex specifically, among several other DMN nodes, in cognitively normal individuals with amyloid pathology (Sheline et al., 2010). These findings were then replicated in *APOE* ε4 carriers without any cognitive impairments and prior to the onset of amyloid pathology, where ε4 carriers had lower RSFC of the precuneus to bilateral hippocampus and left parahippocampus compared to non-carriers (Sheline et al., 2010). While we did not find a main effect of *APOE* ε4 status on RSFC, several studies have previously found reduced RSFC within the same areas that we saw a relationship between *APOE*, visuomotor performance, and RSFC. Specifically, they reported reduced RSFC in the posterior DMN in *APOE* ε4 carriers compared to non-carriers, including in the precuneus (Fleisher et al., 2009;Patel et al., 2013; Su et al., 2017), posterior cingulate cortex (Patel et al., 2013; Su et al., 2017), and between the pCun/PCC and hippocampus (Heise et al., 2014). Decreased functional connectivity in the angular gyrus (a region of the IPL) was also previously shown in *APOE* ε4 carriers compared to non-carriers (Wu et al., 2016). Patients with mild cognitive impairment who later develop AD, compared to mild cognitive impairment patients who remain stable in follow-up, also demonstrate significantly decreased RSFC in bilateral IPL (Li et al., 2016).

Additionally, lower RSFC in the DAN was also a predictor of worse non-standard visuomotor performance in APOE ε4 carriers. The DAN, composed bilaterally of the intraparietal sulcus and frontal eye fields, is involved in goal-driven “top-down” orienting of attention toward behaviorally relevant stimuli (Fox et al., 2006). The influence of the ε4 allele on the DAN is not widely studied (Foo et al., 2020). One paper looking at structural MRI found cortical thinning in APOE ε4 carriers in the frontoparietal regions that form DAN functional nodes (Wolk and Dickerson, 2010). Most studies on the DAN and its relation to AD are from clinical populations with either mild cognitive impairment or dementia, demonstrating that “top-down” attention processing mediated by the DAN is impaired by mild cognitive impairment and AD with decreased RSFC observed in both populations (Li et al., 2012; Qian et al., 2015; Zhang et al., 2015; Mcdonough et al., 2019; Wu et al., 2022).

There are several brain regions that are activated during non-standard visuomotor movements, with the precuneus and IPL in particular appearing to be crucial for discriminating between standard and non-standard task conditions (Gorbet and Sergio, 2016, 2018). Other studies have further demonstrated the importance of the IPL in visuomotor control (Bernier and Grafton, 2010; Mutha et al., 2011; Crottaz-Herbette et al., 2014). Regions of the medial temporal lobe, including the hippocampus, parahippocampus, and entorhinal cortex, are involved in encoding hand position in space, and the parahippocampal cortex may be particularly important for sensorimotor transformations between visual inputs and hand kinematics (Tankus and Fried, 2012). The involvement of these regions in visuomotor integration may therefore explain the reduced RSFC that we see between several regions of the DMN including the pCun/PCC, IPL, and parahippocampal cortex in ε4 carriers associated with deficits in non-standard visuomotor movements. Further, the intraparietal sulcus of the DAN is located within the PPC, which is activated during visuomotor transformations (Blangero et al., 2009). Taken together, our findings from the DMN and DAN demonstrate a relationship between parietal–frontal and parietal–temporal functional connectivity and visuomotor performance. This is consistent with the important role of reciprocal cortical networks involving frontal, parietal, and temporal regions in visuomotor transformations required for successful execution of goal-directed movements (Gorbet et al., 2004; Gorbet and Sergio, 2016, 2018; Caminiti et al., 2017).

### Potential mechanisms of *APOE* ε4 effects on resting state functional connectivity

The *APOE* ε4 allele has been proposed to reduce protection or increase toxicity compared to the e3 and e2 alleles in most AD pathogenic pathways. One potential mechanism of *APOE* ε4 action is *via* Aβ-induced neurotoxicity (Mahley and Rall, 2000; Manelli et al., 2004). Histopathological studies demonstrated that *APOE* ε4 carriers have a higher Aβ plaque density and burden compared to non-carriers (William Rebeck et al., 1993; Tiraboschi et al., 2004; Reiman et al., 2009; Caselli et al., 2010). In cognitively normal individuals, biomarkers of Aβ accumulation were present at a frequency that corresponded to their *APOE* genotype, with the highest frequency found in *APOE* ε4 carriers (Castellano et al., 2011). The *APOE* E4 protein appears to bind with less affinity to Aβ (LaDu et al., 1994), and may therefore prevent effective clearance of neurotoxic Aβ species compared to other *APOE* variants (LaDu et al., 1997). Indeed, Aβ complexes formed with the E2 and E3 proteins are cleared more rapidly from the brain compared to complexes with the E4 variant (Deane et al., 2008). Several studies have demonstrated decreased functional connectivity in the DMN in cognitively normal subjects with high amyloid burden (Hedden et al., 2009; Sperling et al., 2009; Sheline et al., 2010; Mormino et al., 2011). Multiple studies have found a general spatial–temporal pattern of amyloid deposition in AD with earliest accumulation in the medial frontal and cingulate regions (Fantoni et al., 2020), which overlap with the core functional nodes of the DMN (Buckner et al., 2008; Fransson and Marrelec, 2008; Andrews-Hanna et al., 2010; Utevsky et al., 2014). A recent study investigating the effects of *APOE* ε4 on Aβ spatial distribution patterns found highest amyloid accumulation in the medial temporal lobe (hippocampal, parahippocampal, and entorhinal cortices) and inferior parietal regions (Cacciaglia et al., 2022). These are some of the same regions between which we reported lower RSFC that predicted poorer visuomotor performance in ε4 carriers. In addition to amyloid plaques, systemic inflammation has been implicated in AD pathogenesis with activation of inflammatory responses observed in the brains of AD patients (Bales et al., 2000). *APOE* may modulate the neuroinflammatory response in AD pathology, as the ε4 allele has been associated with a greater pro-inflammatory response compared to the e3 allele (Guo et al., 2004). Lower RSFC in the DMN and DAN was linked to peripheral pro-inflammatory signaling in older adults without dementia, and was further magnified in *APOE* ε4 carriers (Walker et al., 2020). Therefore, the *APOE* ε4 allele may compromise the functional integrity of resting cortical networks through both Aβ-related and non-Aβ related pathways early in disease progression.

In the current study, while there were no RSFC differences between *APOE* ε4 carriers and non-carriers, the ε4 allele did modify the relationship between visuomotor performance and functional connectivity within the DMN and the DAN. This discrepancy with previous literature showing reduced RSFC in ε4 carriers may be due to sample size differences between the carrier and non-carrier groups. The findings here showed that in *APOE* ε4 carriers, a lower RSFC between posterior functional DMN nodes as well as within the DAN was predictive of impaired task performance. This interaction between genetic risk and RSFC on behavioral performance that is not seen in RSFC alone may point to disruptions to multiple neural mechanisms in *APOE* ε4 carriers that are reflected by impaired performance. A behavioral task that relies on intact integrity of both functional and structural brain networks may be more sensitive to combined disruptions of these networks than either imaging modality alone.

### General conclusion

Sensory and motor regions in the brain are affected by AD pathology, and sensorimotor deficits may precede the onset of cognitive symptoms of AD by several years (Albers et al., 2015). These findings raise the possibility that sensory and motor changes may be an early non-invasive biomarker for AD, and even a target for intervention in the treatment of AD (de Boer et al., 2018; Echlin et al., 2020). Our findings support the potential use of a simple behavioral task that requires communication across brain regions processing visual, cognitive, and motor information to detect sensorimotor (more specifically, visuomotor) impairments. The visuomotor paradigm requiring cognitive rule integration used in the current study may be sensitive to the presence of the *APOE* ε4 allele, the greatest genetic risk factor for AD (Farrer et al., 1997; Bekris et al., 2010; Karch and Goate, 2015). Such a behavioral testing approach could have potential for detecting sensory and motor changes that are specific to AD in the preclinical stage. While the generalizability of these findings is limited by the relatively small sample size and the study being cross-sectional in nature, they provide important insights into the relationship between visuomotor dysfunctions and preclinical AD. With cross-sectional data, it is difficult to determine whether the results in the present study reflect a change in RSFC possibly due to *APOE* ε4 mechanisms, or if they reflect long-standing differences where *APOE* ε4 carriers may have always had lower RSFC that correlates with poorer visuomotor performance. Future work should investigate if individuals demonstrating visuomotor impairments on a non-standard task prior to cognitive decline are more likely to then develop mild cognitive impairment or dementia later in life.

## Data availability statement

The raw data supporting the conclusions of this article will be made available by the authors, without undue reservation.

## Ethics statement

The studies involving human participants were reviewed and approved by Human Participants Review Sub-Committee of York University’s Ethics Review Board. The patients/participants provided their written informed consent to participate in this study.

## Author contributions

AR: conceptualization, methodology, software, formal analysis, investigation, data curation, writing – original draft, writing – review and editing, and visualization. DG: conceptualization, methodology, software, formal analysis, resources, data curation, and writing – review and editing. KH: conceptualization, methodology, and investigation. LS: conceptualization, methodology, formal analysis, resources, writing – review and editing, supervision, project administration, and funding acquisition. All authors contributed to the article and approved the submitted version.

## Funding

This work was supported by a Canadian Institutes of Health Research operating grant (grant number MOP-125915 to LS).

## Conflict of interest

The authors declare that the research was conducted in the absence of any commercial or financial relationships that could be construed as a potential conflict of interest.

## Publisher’s note

All claims expressed in this article are solely those of the authors and do not necessarily represent those of their affiliated organizations, or those of the publisher, the editors and the reviewers. Any product that may be evaluated in this article, or claim that may be made by its manufacturer, is not guaranteed or endorsed by the publisher.
